# Fibroepithelial polyp of the ureter: the value of magnetic resonance imaging of the urinary tract in diagnosis and therapeutic planning

**DOI:** 10.1590/0100-3984.2017.0214

**Published:** 2019

**Authors:** Tiago Kojun Tibana, Rômulo Florêncio Tristão Santos, Luiz Augusto Morelli Said, Edson Marchiori, Thiago Franchi Nunes

**Affiliations:** 1 Universidade Federal de Mato Grosso do Sul (UFMS), Campo Grande, MS, Brazil.; 2 Hospital Regional de Mato Grosso do Sul, Campo Grande, MS, Brazil.; 3 Universidade Federal do Rio de Janeiro (UFRJ), Rio de Janeiro, RJ. Brazil.

**Figure 1:**
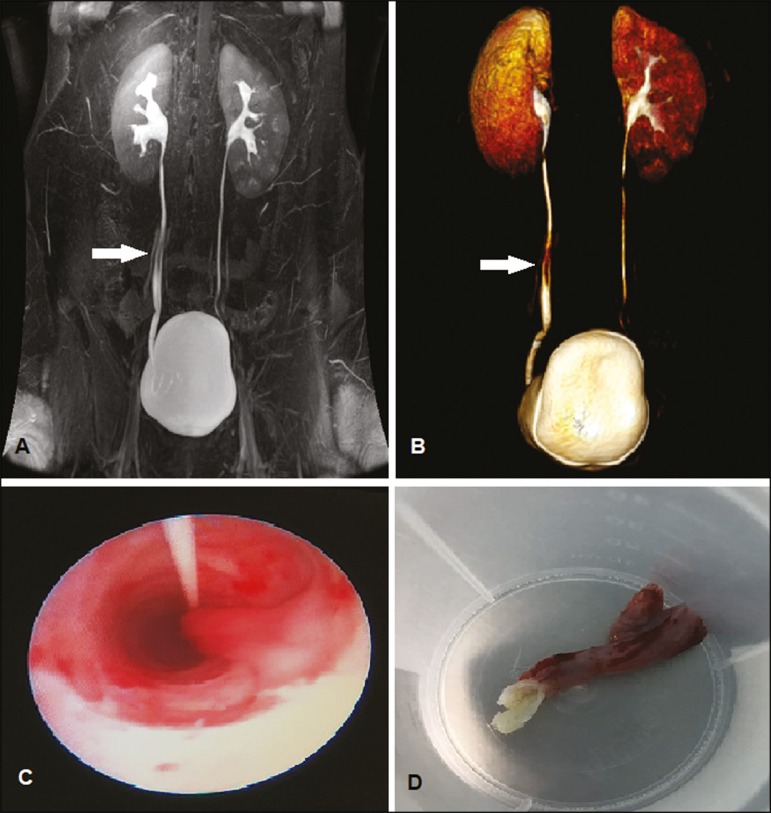
Coronal magnetic resonance imaging of the urinary tract (**A**) and three-dimensional reconstruction (**B**), showing an elongated polypoid formation with a probable origin in the middle ureter (arrow). **C:** Ureteroscopy showing an intraluminal polyp. **D:** Macroscopic aspect of the lesion.

Dear Editor,

A 33-year-old woman presented with a five-month history of intermittent lumbar pain radiating to the suprapubic region. She reported no dysuria or hematuria. Computed tomography showed ureterolithiasis, and the patient was treated conservatively, which resulted in partial improvement. She evolved to worsening of the intensity and frequency of pain, together with pollakiuria. Physical examination revealed no significant alterations. A rapid urine test demonstrated erythrocytes in the urinary sediment. Magnetic resonance imaging revealed an elongated polypoid formation, likely originating from the middle ureter, with inferior displacement, measuring approximately 4.8 cm in length (Figures 1A and 1B). Ureteroscopy showed an intraluminal ureteral polyp (Figure 1C). The patient underwent endoscopic resection (Figure 1D), which was successful, resulting in improvement of the signs and symptoms. The pathology report confirmed the presumed diagnosis of fibroepithelial polyp (FEP).

Although tumors of the genitourinary tract are not uncommon^(^^1^^-^^4^^)^, primary tumors of the ureter are rare, accounting for only 1% of all tumors of the upper urinary tract. Benign lesions are even rarer, accounting for only 20% of all tumors of the ureter, and can be of epithelial or non-epithelial origin. Non-epithelial tumors originate in the mesoderm and include fibromas, leiomyomas, neurofibromas, hemangiomas, and FEP^(^^5^^)^. Although rare, FEPs are the most common benign lesions of the ureter. They are mesodermal lesions consisting of hyperplastic connective tissue with vascular stroma and covered by urothelium. Although the etiology of FEPs is unknown, it is believed that they are slow-growing congenital lesions or result from chronic urothelial irritation caused by inflammation, infection, trauma, or obstruction. They are more common men, at a ratio of 3:2, most are solitary lesions, and most are less than 5 cm in length^(^^6^^,^^7^^)^. Hematuria is the most common symptom, although an FEP can manifest as low back pain or, less frequently, dysuria and pollakiuria.

FEPs have a highly variable presentation and can be evaluated using various imaging techniques, which facilitate the localization and diagnosis of the lesion. Intravenous urography and retrograde ureterography are the main imaging modalities employed in the evaluation of a ureteral lesion^(^^5^^)^. Because of the development of faster sequencing techniques, magnetic resonance imaging has been used with increasing frequency, having a number of benefits, such as allowing multiplanar imaging, providing excellent soft tissue contrast, and not exposing patients to ionizing radiation. It can delineate the extent of the tumor, providing important information for therapeutic planning and for making a more accurate diagnosis. When the imaging shows that there is no local invasion, regional lymph node involvement, or distant metastases, it supports a diagnosis of benign ureteral lesion. FEPs typically appear as thin, elongated, generally smooth filling defects that are often found in the proximal ureter and are sometimes accompanied by ureterohydronephrosis^(^^5^^)^. The presence of urine around the filling defect, a polypoid outgrowth, and a long ureteral mass are imaging features highly suggestive of FEP^(^^7^^,^^8^^)^. Histological confirmation should always be obtained before definitive treatment is administered^(^^6^^)^.

Although the treatment of choice is minimally invasive local resection, it is not uncommon for segmental ureterectomy or nephroureterectomy to be performed when there is uncertainty in the preoperative diagnosis. In the case of renal exclusion due to prolonged obstruction, the treatment of choice is nephroureterectomy^(^^9^^,^^10^^)^.
